# The saga of the many studies wrongly associating mitochondrial DNA with breast cancer

**DOI:** 10.1186/1471-2407-14-659

**Published:** 2014-09-09

**Authors:** Antonio Salas, Manuel García-Magariños, Ian Logan, Hans-Jürgen Bandelt

**Affiliations:** Unidade de Xenética, Instituto de Medicina Legal, and Departamento de Anatomía Patolóxica e Ciencias Forenses, Facultad de Medicina, Universidad de Santiago de Compostela, 15782 Galicia, Spain; Departamento de Matemáticas, Universidade da Coruña, A Coruña, Spain; Exmouth, Devon, UK; Department of Mathematics, University of Hamburg, 20146 Hamburg, Germany

**Keywords:** Epistasis, SNP-SNP interaction, Complex disease, Association study, mtDNA, Haplogroup

## Abstract

**Background:**

A large body of genetic research has focused on the potential role that mitochondrial DNA (mtDNA) variants might play on the predisposition to common and complex (multi-factorial) diseases. It has been argued however that many of these studies could be inconclusive due to artifacts related to genotyping errors or inadequate design.

**Methods:**

Analyses of the data published in case–control breast cancer association studies have been performed using a phylogenetic-based approach. Variation observed in these studies has been interpreted in the light of data available on public resources, which now include over >27,000 complete mitochondrial sequences and the worldwide phylogeny determined by these mitogenomes. Complementary analyses were carried out using public datasets of partial mtDNA sequences, mainly corresponding to control-region segments.

**Results:**

By way of example, we show here another kind of fallacy in these medical studies, namely, the phenomenon of SNP-SNP interaction wrongly applied to haploid data in a breast cancer study. We also reassessed the mutually conflicting studies suggesting some functional role of the non-synonymous polymorphism m.10398A > G (ND3 subunit of mitochondrial complex I) in breast cancer. In some studies, control groups were employed that showed an extremely odd haplogroup frequency spectrum compared to comparable information from much larger databases. Moreover, the use of inappropriate statistics signaled spurious “significance” in several instances.

**Conclusions:**

Every case–control study should come under scrutiny in regard to the plausibility of the control-group data presented and appropriateness of the statistical methods employed; and this is best done before potential publication.

## Background

Studies on mitochondrial DNA (mtDNA) in human disease have often been intensely debated (see some examples on cancer instability [1–3]). On the one hand, mtDNA case–control association studies are frequently affected by several problems related to deficient study designs and inappropriate statistical methods [4, 5]. On the other hand, a phylogenetic approach has proven to be extremely useful in discovering various kinds of errors in these studies, which has often compromised their results and conclusions [1, 6–9]. The allelic view [1] on mtDNA variation (as haplotypic variation) is also a common misconception in mtDNA studies, where single variants from haplogroup motifs are treated as if they were potentially independent disease markers. A new manifestation of this problem has to do with the phenomenon of SNP-SNP interaction applied to the interpretation of mtDNA variation.

Epistasis, a term coined by Bateson [10], was first defined as a masking effect whereby a variant or allele at one locus prevents the variant at another locus from manifesting its effects [11]. Epistasis and genetic interaction refer to the same phenomenon; however, the former is widely used in population genetics and especially refers to the statistical properties of the phenomenon. In essence, SNP-SNP interaction makes sense whenever two SNPs are located in different (unlinked) loci, and it is generally applicable to the sphere of the autosomal genome. Since the whole mtDNA molecule is virtually a single haploid locus, the concept of epistasis or interaction, by definition, does not apply to SNP-SNP interaction of mtDNA variants. Thus, the concept of haplotype is of a different nature; haplotype refers to a combination of tightly linked variants in a segment of a chromosome (which could be a gene, or the entire mtDNA molecule). There are some haplotypes (or more generally, some haplogroups) that have been reported as contributing to the risk of a disease, as it is the case of several of articles discussed in the present study. It is the variants combined in this DNA segment that contribute to the risk as a single locus, but not by way of interaction of independent loci; it is in fact the haplotype as a whole that is used to test for association, not the interaction between the variants that compose this haplotype. However, the term epistasis has been incorrectly used in a few mtDNA studies; for example, in designating the potential effect of a haplotype in cancer, as expressed by Canter et al. [12]: “*epistatic interactions between individual loci within the mitochondrial genome as well as nuclear-mitochondrial gene interactions should be investigated*”.

In the present study we discuss the example [13] where the concept of interaction has been misunderstood and misapplied. This example provides an opportunity to discuss the role of the polymorphism A10398G alias m.10398A > G (Thr > Ala) in breast cancer, which has been the focus of as many as ten studies (and many more reviews) in the period 2005–2013. By critically reviewing all these studies we confirm the conclusion of the meta-analysis performed by Francis et al. [14] that the association results reported to date are contradictory and inconsistent, thus providing absolutely no evidence supporting a role of this mtDNA polymorphism in breast cancer. However, closer examination of every single study that claimed to have found some association reveals that there were clear indications in either the data presented or the statistical methods used that the association was spurious.

## Methods

Phylogenetic methods are indispensable for mtDNA studies of human disease [4]. In particular, we use PhyloTree Build 16 (http://www.phylotree.org/ [15]) and the information provided concerning haplogroup status alongside with direct inspection of the GenBank entries translated into mutation motif lists (relative to the standard reference sequence, the rCRS) available in the Web (http://www.ianlogan.co.uk/sequences_by_group/haplogroup_select.htm; http://www.ianlogan.co.uk/checker/accession.htm). Note however that PhyloTree employs a confusing nomenclature which does not conform to the rCRS based notation used in medical genetics [16]. Here, when focusing on a single variant A or G at 10398, we write A10398 and 10398G, where the prefix position is reserved for the rCRS nucleotide and the suffix for the any other variant, without invoking ancestry [16].

The statistical relationship between single test results (e.g. using Fisher’s exact test for every mtSNP) and logistic regression analysis (applied to the detection of mtSNP-mtSNP interaction) can be examined by way of a simple simulation analysis. For such an experiment, two identical data sets of mtSNP genotypes were taken from the control group in the “mainland Spain” breast cancer series (*N* = 616) reported by Mosquera-Miguel et al. [5]; all the profiles in one sample were now artificially labeled as cases while the other profiles in the other copy sample was labeled as “controls” and stayed unchanged. A mtDNA profile belonging to haplogroup K1 was added one at a time to a maximum of 120 to the subset of “cases” and several statistical analyses were each performed for “controls” *versus* “cases”. The goal of the simulation was to create scenarios where haplogroup K1 is progressively overrepresented in cases compared to controls. A Fisher exact test is performed for the mtSNPs defining haplogroup K1 (10398G, 12308G) and for the amount of haplogroup K1. Logistic regression was also carried out in order to detect the ‘interaction’ between mtSNPs A10398 and 12308G as done in [13].

Computation of statistical power was carried out using mitPower [17]. The computation was done in a conservative manner with the only aim of highlighting those case–control association studies that are extremely underpowered. We did not consider the *a posteriori* power estimation option in mitPower under the assumption that estimates would be strongly affected by the fact that population stratification has severely inflated frequencies in cases *versus* controls in the different cohorts reviewed (see Results below). We instead computed statistical power assuming a ‘*de novo*’ study that considers the reported sample sizes in cases and controls from the different cohorts and the frequency of 10398G in their controls. We assumed a conservative risk (odd ratio) for 10398G equal to 2. Power was computed using Fisher’s Exact Test for the most conservative scenario that considers 2 × 2 tables. Note that generally a threshold of at least 80% is (consensually) considered to be an adequate power in case–control association studies.

## Results

### Negative findings for association of A10398G with breast cancer

Solid association studies would ideally use at least two large independent pairs of case and control cohorts, one employing a test sample and a subsequent replication (validation) sample. There are only four studies on the A10398G polymorphism which have employed more than a single case–control sample, namely the ones by Canter et al. [12], Setiawan et al. [18], Mosquera-Miguel et al. [5], and Francis et al. [14]. The first of these studies claimed some association in only one cohort (but no association in their other two cohorts), which on its own does not permit featuring the polymorphism as being associated with breast cancer, although the authors have decided otherwise and turned this into a story; see below.

Setiawan et al. [18] analyzed three different cohorts of U.S. American patients with their respective controls, and they also carried out a joint analysis of the three cohorts. This represents therefore the largest analysis to date on breast cancer and the A10398G polymorphism (Table 1). They did not find any statistical association in any of the cohorts or the combined cohorts. The study of Mosquera-Miguel et al. [5] analyzed two pairs of Spanish cohorts for various mtDNA polymorphisms and found no association with sporadic breast cancer patients.

**Table 1:** **Summary of the different case–control association studies targeting the mtDNA polymorphism m.10398A > G. statistical power was computed using mitPower**

Reference	Population/Ethnicity	No. of cases	No. of controls	Power (%)	‘Risky’ allele
		(frequency of A10398)	(frequency of A10398)		
[12]	‘African-Americans’	48 (15%)	54 (6%)	15.1	None
	‘African-Americans’	654 (13%)	605 (9%)	98.4	A
	‘Whites’	879 (80%)	760 (79%)	100	None
[13, 19]	Non-Jewish European American	156 (68%)	260 (79%)	66.1	G
[14]	Tamil Nadu; South India	279 (38%)	280 (45%)	97.8	None
	Andhra Pradesh, South India	348 (46%)	352 (45%)	99.2	None
	Karnataka, South India	89 (38%)	92 (42%)	59.1	None
[5]	Spanish mainland	464 (84%)	453 (82%)	94.9	None
	Canary Island (Spain)	302 (76%)	295 (78%)	88.5	None
[20]	North India	124 (57%)	273 (44%)	85.6	A
[18]	‘African-American’	542 (7%)	282 (4%)	49.4	None
	Multi-ethnic cohort	391 (7%)	460 (6%)	78.2	None
	CARE and LIFE cohort	524 (6%)	236 (7%)	72.1	None
[21]	Polish	44 (77%)	100 (97%)	0.1	G
[22]	Southern Chinese	28 (26.9%)	45 (39.5%)	25.3	None
[23]	Bangladesh; India	24 (75%)	20 (35%)	16.7	A
[24]	Iraq	21 (100%)	16 (100%)	11.2	None
	Iraq	21 (100%)	22 (91%)	0	None
[25]	Malay	101 (27%)	90 (46%)	61.4	G

Most recently, Francis et al. [14] analyzed a large cohort of cases and controls in three Dravidian populations from South India. They also carried out a meta-analysis of 16 groups (from published sources and including the three novel groups), thus comprising a very large number of cases and controls from various populations. Overall, the results show no association of the A10398G polymorphism and breast cancer risk.

### SNP-SNP interaction and haplogroup association: using the same data twice

Covarrubias et al. [13] analyzed a cohort of patients suffering with breast cancer and a control group of healthy subjects in order to investigate the presumable association of mtDNA SNP-SNP interaction with the disease. According to the authors, the main finding of their study was the detection of a highly significant interaction between the variants 12308G and 10398G, with results suggesting that these variants increase the risk of a woman developing breast cancer.

From that article one can immediately infer that these authors have used exactly the same case and control samples as employed in their previous article [19]. Instead of explicitly telling the reader that they have done so, the authors first described their sample by saying that “s*ixty-nine mtDNA variants were genotyped in DNA samples from 156 non-Jewish European American breast cancer patients and 260 ethnically age matched female controls…* ” [13]. When one further learns that “a*dditional details on subject ascertainment, mtDNA genotyping, and initial analysis can be found in Bai* et al. *(2007)*” and compares the information about the sample collection in both studies, one can finally infer that the group of subjects analyzed in both studies must have been exactly the same. Moreover, although not clearly stated, Covarrubias et al. [13] used a subset of the genotyping data employed before; in fact, their Table one is a brief replicate of Table two in Bai et al. [19]. Hence, the findings reported by Covarrubias et al. [13] cannot be considered as independent evidence of an association of mtDNA variants with breast cancer, but at best as an attempt at a reanalysis using logistic regression.

In the Bai et al. [19] study, the main finding was that carriers of haplogroup K showed an increased risk of suffering sporadic breast cancer (OR = 3.03; 95%; CI 1.63-5.63). In reality, the authors targeted the slightly larger haplogroup U8b by using the variants 9055A and 12308G for identification. On the other hand, the main finding of the Covarrubias et al. [13] study was that “*a highly significant interaction was identified between variants 12308G and 10398G (empirical P value = 0.0028), with results suggesting these variants increase the risk of a woman developing breast cancer (OR = 3.03; 95% CI 1.53-6.11)*”. Not surprisingly, the variants 10398G and 12308G together define the main European branch of haplogroup K, namely, K1 (which seems to make up about 80-90% of haplogroup U8b or K in most of Europe). Comparing Figure two of Bai et al. with the current tree presented in PhyloTree, one can infer that as many as 42 out of 47 samples assignable to haplogroup U8b belong to haplogroup K1. Then the frequencies of K1 in cases and controls can be inferred as 27/156 (17.3%) and 15/260 (5.8%), respectively. In other words, the results reported in both studies, though using different terminology, equally reflect the overrepresentation of haplogroup K1 in cases compared to controls.

Statistical interactions can obviously arise under a logistic regression test when analyzing SNPs that are in strong linkage disequilibrium (as is the case with the variation along the whole mtDNA genome). This, however, does not necessarily have to be interpreted as an interaction *per se* [26] but rather as two (or more) SNP variants that predominantly occur simultaneously within the subhaplogroup they define.

**Figure 1:**
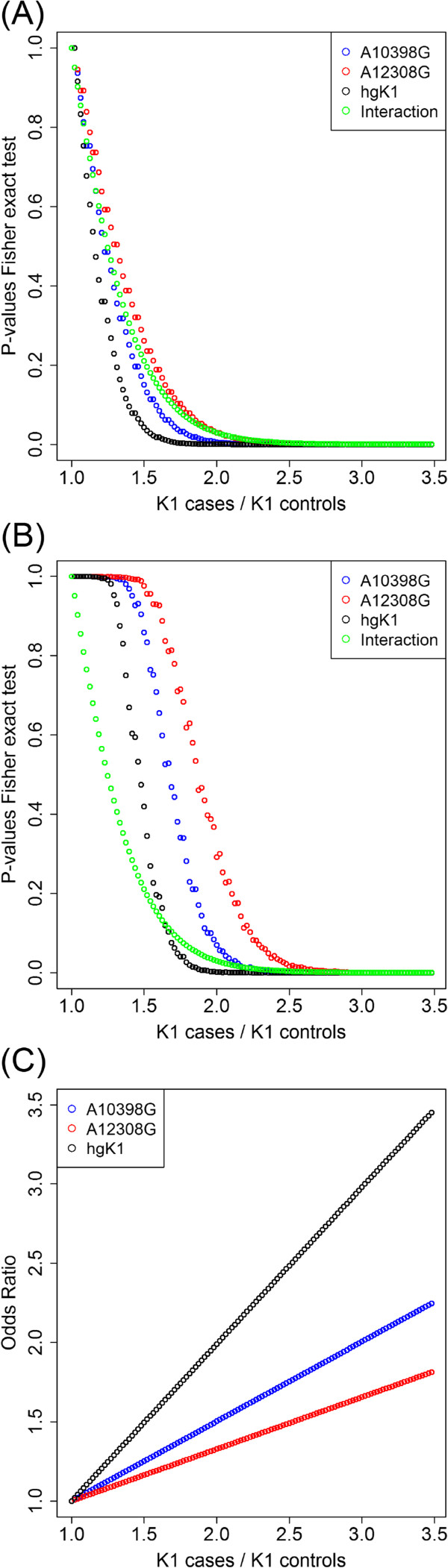
**Statistical tests between artificial cases and controls in a simulation-based approach, where the amount of haplogroup K1 mtDNA profiles in cases is progressively increased with respect to controls. (A)** *P*-values; **(B)** adjusted *P*-values (based on a permutation procedure); **(C)** odds ratio (OR) values. *P*-values are reported for (i) the Fisher exact test applied to mtSNP 10398G, 12308G, and haplogroup K1; and (ii) for the logistic regression interaction between mtSNPs 10398G and 12308G. The permutation analysis was not carried out in the interaction scenario (Figure 1B) because multiple test correction is not necessary as only a single test is carried out at the time.

The statistical effect of this seeming interaction can be studied by using a simulation (see Methods). As shown in Figure 1, there is a high correlation (*r*^2^ > 0.93) between the statistical significance values (based on Fisher exact test) obtained when comparing the proportion of 10398G, 12308G, and haplogroup K1 in cases with respect to controls (that is, the scenario of [15]). These *P*-values are also strongly correlated (*r*^2^ > 0.95) with those obtained for the ‘pseudo-interaction’ between mtSNPs 10398G and 12308G using logistic regression, that is, the scenario of Covarrubias et al. [13].

### Selecting the most convenient statistical approach to generate some positive finding

In the study by Fang et al. [22] two sources of questionable choices of statistics are evident. These authors studied breast cancer and two other kinds of cancer in cohorts of patients from southern China. The basic distinction they made was between haplogroups M and N. They found haplogroup M in 69/104 (66.3%) of the cases but in only 60/114 (52.6%) of the controls. The *P*-value of the two-tailed exact Fisher test for the corresponding 2 × 2 contingency table is 0.0532, thus this could only be considered as marginally significant if assuming a nominal significant value of 0.05. However, the authors preferred to use the two-tailed chi-square test in this case: the uncorrected *P*-value for the chi-square test equals 0.0396, thus seemingly significant. However when employing the recommended Yates correction (see e.g. http://graphpad.com/quickcalcs/contingency1/) the corrected *P*-value (0.0549) would have come much closer to the exact value. In theory, Fisher’s exact test in such a context should always be employed as long as sizes of the samples permit this because this test is valid for all sample sizes. A chi-square test is acceptable only with very large sample sizes, bearing in mind that the significance values it provides always constitute some approximation. The use of a chi-square test thus cannot be justified in Fang’s et al. [22] study because the sample sizes were quite small there. A chi-square test is favored in large-scale genome-wide association studies in part for computational reasons, and this is well accepted by the scientific community. The decision of using a chi-square or a Fisher’s exact test is irrelevant most of the time as both point in the same direction. But the problem comes when the chi-square test is chosen instead of the exact test solely for the purpose of attaining ‘significance’ which could not have been achieved with the exact test.

Since virtually all haplogroup M lineages bear 10398G, whereas in haplogroup N the frequency of 10398G is only about 10-20%, one can expect that in a southern Chinese population a relative excess of haplogroup M would correlate with some higher percentage of 10398G in a sample. And indeed, in that case–control study we are seeing a 10398G frequency of 76/104 (73.1%) in cases and 69/114 (60.5%) in controls. The *P*-value for the two-tailed exact Fisher test is 0.0618. The corresponding uncorrected chi square test delivers a *P*-value of 0.0499 but with Yates correction 0.0691, again closer to the exact value. Fang et al. [22] have again chosen to use only the uncorrected chi-square test.

Furthermore, Fang et al. [22] selected one subhaplogroup (D5) of haplogroup M out of several candidates (D4a, D4, D5, G, M7, M8, M10a) that showed a particularly contrasting frequency between cases and controls. The authors did not attempt to correct for the multiple hypotheses (haplogroups in this case) tested, which would yield non-significant *P*-values for all the haplogroups tested. Last but not least, no validation cohort was offered for the seeming association. Therefore this study failed to demonstrate any association of mtDNA haplogroups with breast cancer because of using inappropriate statistical tools and deficient design.

### The questionable role of A10398G in breast cancer

#### The use of confusing nomenclature

The rCRS [27] nucleotide at position 10398 is A. Therefore the correct notation for the transition at this position is A10398G or m.10398A > G following the official nomenclature in medicine.

In their breast cancer paper, Canter et al. [12] erroneously employed the notation “G10398A”, which incidentally could be interpreted *post hoc* as if it followed the evolutionary order of nucleotide change (from the ancestral G to a first derived A at 10398), but this was certainly not intended by the authors since C7028T, C14766T, T16189C, and T16519C all follow the standard rCRS-based style. The incorrect and confusing notation “G10398A” was then repeatedly used in numerous follow-up papers [20]. This led to the most paradoxical situation that “G10398A” has become more widespread in use within the past five years than A10398G: e.g. Google now has ~60,600 entries for ‘G10398A mtDNA’ but only ~3010 entries for ‘A10398G mtDNA’. This cannot be explained by the 2012 switch from A10398G to “G10398A” executed in PhyloTree since this equally transformed the notation for the C7028T polymorphism by reversing the roles of C and T: there are ~4250 entries for the standard form ‘C7028T mtDNA’ but only ~355 entries for ‘T7028C mtDNA’.

The confusion that has set in with the erroneous designation of the A10398G polymorphism is best reflected by a brief comment on the Canter et al. [12] study given by Benn et al. [28], where it is stated that “*The mt10398a > g polymorphism present in European haplogroups J, K, and Z has been associated with increased risk of invasive breast cancer in black women (48 cases/54 controls and validated in 654 cases/605 controls) but not in white women (879 cases/760 controls).*” Leaving aside the inaccuracies concerning haplogroups K (which as a whole is not defined by 10398G) and Z (not of European ancestry), the Canter et al. [12] study was misinterpreted: first, the smallest case–control analysis did not reach significant values (see below), so that the next one could not be regarded as a validation of the former; second, the nucleotide states A and G for the claimed association were confounded.

Covarrubias et al. [13] claimed that “o*ne of the interactions, 4216C and 10398G, observed in this* [their] *study although not statistically significant after controlling the GWER (Table two), was previously reported in the Canter* et al. *case–control study…*” This affirmation was unfortunate, because it was the rCRS nucleotide A10398 that was reported as being associated with breast cancer by Canter et al. Further, the authors mentioned correctly (in regard to the Canter et al. study), that “*a synergistic interaction was observed between 4216C and 10398A*”.

The G nucleotide is the ultimately ancestral nucleotide at 10398 with respect to the entire (known) mtDNA human phylogeny, while the A nucleotide is considered to be the derived allele. However, it is important to note that this site mutates as many as 21 times (5 from G to A and 16 from A to G) in the basal classification tree, Phylotree Build 16, pointing to independent mutational events occurring at different times. The age of the mutation therefore varies depending on the targeted branch (haplogroup) in the phylogeny. The fact of being ancestral or derived could be completely irrelevant here from the point of view of its presumable pathogenicity (there seems to be a bias towards considering ‘ancestral’ alleles as generally healthy and the ‘derived’ ones as potentially pathogenic). It could be the case that the seeming ‘ancestral’ nucleotide is in fact more recent than the seeming ‘derived’ allele if one focuses on a particular mtDNA haplogroup where this polymorphism has mutated back to the ancestral nucleotide several times. The concept of ancestral allele is also misunderstood in the literature. For instance, Czarnecka et al. [21] mentioned that “w*hile the revised Cambridge reference sequence* [14] *lists the wild type base as A, the alternate base (G) is also prevalent in many populations”*. This affirmation reflects a popular misconception of the rCRS as being the ‘wild-type’ sequence; in reality, the rCRS represents just one particular European mtDNA sequence used for notational purposes [16]. All existing mtDNA lineages are quite distant from the root of the entire mtDNA tree.

Therefore, one has to be aware that the A10398G polymorphism targeted in different studies does not necessarily refer to the same mutational events, and therefore, different studies could in reality be referring to different statistical associations. Thus, for instance, the statistical association could have been found in combination with another variant, then pointing to a particular haplogroup and not to the complex polyphyletic group defined by a particular nucleotide at 10398.

The reply of Bai et al. [19] to the Mosquera-Miguel’s et al. [5] article testifies to a basic misconception of the role of haplogroups in disease studies: “*Although haplogroup K is a subclade of haplogroup U in Europeans, most, if not all published articles on haplogroups and disease association do not include haplogroup K within haplogroup U*”. Solely mutations that define monophyletic clades could be pinpointed to generate an effect on the fitness of the mtDNA. U minus K is just a conglomerate of nine monophyletic clades. Unfortunately, Bai et al. were correct in claiming that most medical geneticists perceive haplogroup U as an entity that does not include haplogroup K – but a mistake remains a mistake whether it is committed by a majority of researchers or not. This U-K misconception has its root in an early article on the classification of European mtDNA [29], which was based on limited mtDNA information as provided by RFLP analysis at the time.

#### The conflicting signals in the studies of Canter et al. and Bai et al

Canter et al. [12] analyzed three different cohorts of ‘African-American’ women with invasive breast cancer. In their pilot study (48 cases and 54 controls; all ‘African-American women), the authors did not find any association of this polymorphism with the disease.

In a second ‘African American’ cohort (654 cases, 605 controls) these authors found the A10398 nucleotide significantly increased in breast cancer patients. In a third cohort of ‘White’ women (879 cases, 760 controls) they did not detect any statistical association. The authors understand their findings as a “*novel epidemiologic evidence that the mtDNA 10398A allele influences breast cancer susceptibility in African-American women”.*

In reply to an article by Mims et al. [30] on prostate cancer (see also Verma et al. [31]), the response by Canter et al. [26] provided us with more clues about their previous findings from 2005 [12] (since they used the same breast cancer cohorts as in their 2005 study). Thus, one can infer that the main association signal found by Canter et al. [12] appears due to the combination of the variants A10398 and 4216C. These two variants together are good markers for the European haplogroups J1c8, T, and R2 (see PhyloTree) with haplogroup T being the most prevalent one. In other words, the statistical signal reported by Canter et al. in [12, 32] just mirrors the existence of an increased component of matrilineal European ancestry in their cases compared to their controls.

Therefore, there is little support to the positive association reported in the Canter et al. study if we consider that the phylogenetic evidence clearly points to a false positive due to the confounding effect of population stratification. Control for the latter should be mandatory in case–control studies in order to avoid false positives, especially when targeting ‘African-Americans’ for which one might *a priori* suspect large differential matrilineal admixture proportions in cases and controls [33].

In contrast to the Canter studies, Bai et al. [19] and the follow-up (non-independent) Covarrubias et al. [13] study led to the conclusion that it is the 10398G variant that would be related to an increase of risk to suffer breast cancer. Considering the SNPs targeted by Bai et al., the variant 10398G pointed mainly to haplogroup K1 in their cases and controls (see e.g. their Figure two).

Covarrubias et al. [13] reported an interaction between 10398G and 12308G, (thereby ‘recycling’ the same findings reported by Bai et al.), thus effectively claiming that K1 has an apparent statistical association with breast cancer. Note therefore that the Canter’s implicit finding (the ‘risky’ haplogroup T) was not replicated in Covarrubias et al. [13] while *vice versa* the Covarrubias et al. [13] finding (concerning the ‘risky’ haplogroup K1) was not observed in Canter’s study.

#### The risk of the 10398G variant in polish breast cancer patients

Czarnecka et al. [21] reported the association of 10398G in a Polish breast cancer cohort (44 cases and 100 controls). Apart from being an underpowered case–control study (Table 1), population stratification was not monitored. Regarding the latter, it is important to mention that stratification has to be measured empirically, and that the fact that controls “*…matched for ethnicity and region of residence.*” does not guarantee lack of stratification. There are in fact solid reasons to believe that this study suffered from a strong bias in the estimation of the frequency of the 10398G mutation in controls. Czarnecka et al. [21] reported 10398G in 10 (23%) of the 44 cases (from one medical center) and 3 (3%) of the 100 controls (which came from a geographically distant medical center). Fisher’s (2-sided) exact test delivers an extraordinarily low *P*-value of 0.00042 for this contingency table. It is surprising that no reference had been made to four or five sets of Polish mtDNA population data of total size >3,000, which were available in September 2008, well before the submission of that article. No attempt was made to compare the in-house control-region data with any one of these data sets, although one of them [34] was taken as the major part of the control group in a publication submitted two months later [35].

In any European population there are three major haplogroups defined by 10398G: haplogroups J, K1, and N1a1. Using control-region data, one can reliably recognize the following haplogroups by minimal motifs: J (16069T-16126C), K (16224C-16311C) *versus* K1a (16224C-16311C-497T) and K1c (16224C-16311C-498del), and haplogroup N1a1b (250C alone or plus 16391A) in which subhaplogroup I is nested as its dominating component. One thus loses N1a1a but gains J1c8 (with back mutated A10398), both of which are very minor and expected to be equally uncommon (<0.3%). In an enlarged Polish control group of 414 normal individuals, Gaweda-Walerych et al. [36] detected 22 carriers of haplogroup K, including 15 of its subhaplogroup K1a and 2 of K1c. Hence 17/22 > ¾ is a conservative estimate of the K1 proportion of K. When applying this ¾ rule for estimating the K1 frequencies in different samples, we obtain compound frequency of 117/894 (13.1%) for N1a1b, J, and K1 in the three Polish data sets stored in EMPOP (http://www.empop.org) and frequency of 47/277 (17.0%) for I, J, and K1 for the control group in Gaweda-Walerych et al. [36]. In total, we thus obtain the (conservative) frequency estimate 164/1171 (14.0%) for the occurrence of 10398G in Poland. This is also well in agreement with an estimate derived from the Polish haplogroup frequencies of Piechota et al. [37], who effectively targeted haplogroups I, J, and U8b (instead of the claimed K): assuming a lower bound of ^2^/_3_ for the K1 contribution to U8b, one obtains the estimate of 23/152 (15.1%) for 10398G in Poland. This data set served as the minor part for the control group of Czarnecka et al. [35]. Incidentally, the still larger Polish data set of Saxena et al. [38] would give an estimate of 13.0% using the same method of estimation. However, in that article one can directly read off the real frequency of 10398G in this Polish data set, viz. as 337/2006 (16.8%), which demonstrates that our estimation was even somewhat too conservative.

We then performed four (2-sided) exact Fisher tests for the control and cases data from Czarnecka et al. [35] each compared against either of the literature data sets with counts 164 vs 1007 and 337 vs 1669, respectively. The control data from their study receive *P*-values of 0.00059 and 0.00004 (sic!) in these comparisons. This demonstrates that their control group either (1) is so special that it would have been mandatory to monitor population stratification village by village or (2) the genotyping went wrong quite badly or (3) the samples had not been chosen and aggregated in a correct way. Therefore, it is evident that the control group employed by Czarnecka et al. [35] cannot represent the population of cases and yield nucleotide variant frequencies that are completely unexpected in view of the patterns observed in other data sets. Comparing the frequencies for the cases in that paper to either of the literature data we get *P*-values as high as 0.122 and 0.309, very far from being significant.

#### The risk of the A10398 variant in Indian breast cancer patients

Darvishi et al. [20] analyzed 124 breast cancer patients and 273 controls; the authors reported a statistical association of A10398 with breast cancer. Given the fact that these authors targeted an Indian population and that they only genotyped the A10398G polymorphism, one has to assume that the main statistical signal came from haplogroup N as a whole (10398 is one of the mutations that separates the two (macro)-haplogroups M and N in Asia). By way of analyzing squamous cell carcinoma samples (55 cases *versus* 163 controls), they also reported a positive association for A10398.

The frequency of haplogroup N in India is highly heterogeneous, as even highlighted by Darvishi et al. [20]. Their cases and controls were selected from North India (without reporting any further geographic specification). It is noteworthy that their control sample has a frequency of A10398 of 43.6% (compared to 57.3% in their cases); however, the frequency of haplogroup N in e.g. Gujarat (Northeast India) and Punjab and Kashmir (North India) is just below 60% as also reported by the same authors (see their Figure two), thus nearly matching the frequency of haplogroup N in their cases. This means that their control group does not properly represent their cases, and therefore pointing once more to a false positive case of association.

On the other hand, the results of Darvishi et al. [20] enter in conflict with the recent article by Francis et al. [14] carried out on a much larger sample of Indian patients and controls (three different cohorts plus meta-analysis) where they found no association of the A10398G polymorphism and breast cancer.

#### The risk of the A10398 variant in breast cancer patients from Bangladesh

Recently, Sultana et al. [23] analyzed a sample of only 24 breast cancer cases and 20 controls from Bangladesh, claiming the association of A10398 and C10400 with breast cancer. These authors therefore targeted the (macro-haplogroup N). It is surprising to see that the frequency of haplogroup N in their cases is 75% *versus* 25% in controls.

To explain a possible false positive finding in the Sultana et al. [23] study one could easily allege: (i) deficient statistical power due to their extremely small cohort, and (ii) the confounding effect of populations sub-structure (given that these authors have not controlled this possible confounding factor). Their most recent study, Sultana et al. [39] give us more clues about this interesting case example. In the latter study, these authors analyzed exactly the same samples as in their 2011 article [23], but instead of targeting the coding region, they examined now the control region. The authors reported that “*two novel polymorphisms in the D-loop, one at position 16290 (T-ins) and the other at 16293 (A-del), was higher in breast cancer patients than in controls*”. From the sequence electropherogram of their Figure one one discovers that the authors misaligned their sequences with respect to the rCRS: their two indels constitute in fact the well-known transition C16290T and the transversion A16293C. Both resulting variants together signal the rare haplogroup A11 within haplogroup N (PhyloTree) and would therefore necessarily bear the combination A10398-C10400 seen in their 2011 article). This haplogroup status enters in phylogenetic conflict with another mutation that is reported in their 2012 article; the authors mentioned that 10316G is present in 69% of their cases but not in their controls. The transition 10316G is a good marker for haplogroup M43 and R22; but it has not yet been reported within A11. Whatever the solution to this phylogenetic puzzle would be, their data point to the fact that cases carry an exaggerated representation of a rare haplogroup (most likely A11) that constitutes 75% of them, thus inflating the signal given by haplogroup N in their cases. This is another clear demonstration of population stratification or inadequate selection of cases and controls in that small Bangladesh sample. Since the full haplotypes have not been presented in either article, one has to reject the conclusions drawn by the authors.

#### The risk of the 10398G variant in Malaysian breast cancer patients

Nadiah et al. [25] reported the presence of the 10398G variant in 73% of breast cancer patients compared to 54% in controls, both from Malaysia. Their sample sizes are somewhat underpowered: 101 cases and 90 controls. They did not use a replication cohort. The direction of the association reported by these authors adds further noise to the global scenario; it was the G nucleotide that was found to be over-represented in cases (OR = 2.29). To explain such a phenomenon the authors alleged that “*(the differing results may be due to the variability of risk modifiers that exist in diverse geographical areas)*”. It is however difficult to conceive how a risk modifier can completely invert the direction of the risk; it was in fact the A nucleotide that was reported to be associated in other South East Asian populations (e.g. Bangladesh and India).

#### No association of the 10398G variant in Iraqi women

Ismaeel et al. [24] have recently analyzed the 10398G variant in 21 females with breast malignant tumors, 22 females with breast benign tumors and 16 healthy females used as controls. Only two females of the benign tumors group (9%) carried the 10398G variant (then, 0% in their controls and in the breast malignant tumor cohort). The frequency of this variant in these Iraqi females is surprising when comparing with other datasets from the country where this variant could reach 31% [40]. Given the fact that these Iraqi patients and controls seem to represent a typical population from Iraq, it is most likely that some methodological error occurred with the genotyping (based on RFLPs) of these samples.

#### Publication bias in breast cancer risk

Several review articles have been written since the first publication of Canter et al. [12] in regard to the implications of the 10398 polymorphism in breast cancer (together with other cancers); strikingly as many as original research articles [41–47].

Unfortunately, all these surveys rephrase and summarize the conclusions of the original articles without critically investigating the robustness of the evidences. Worse, most of the time, only the positive findings of the literature are highlighted. Since 2009, and according to The Web of Science (http://ip-science.thomsonreuters.com/es/productos/wok/); the studies by Canter et al. [12] and Bai et al. [19] received 109 and 86 citations (query: 28 February 2014), whereas for the same period, the studies of Setiawan et al. [18] and Mosquera-Miguel et al. [5] showing negative association received 23 and 22 citations, respectively. Czarnecka and Bartnik [41] reported that “the first interesting and *widely investigated mtDNA polymorphism in the cancer field was A10398G, first described as causative factor in breast cancer development (50–52)*”. Curiously, note that in the previous quotation, their citation 50 refers to the negative findings of Setiawan et al. [18] but they do not further mention or comment on this article, and the negative findings of Mosquera-Miguel et al. [5] are not cited at all. In the earlier review by Plak et al. [43] neither of the two reports with negative findings were cited. This kind of publication bias is harmful in science because it stimulates future scientific studies in wrong directions and promotes studies suffering from the same deficiencies as the previous ones.

## Discussion

We can suggest several scenarios that might explain a seeming genuine statistical association between mtDNA variants and a particular disease. First, the variant *per se* is fully responsible for the disease (causal variant). Second, the targeted variant is in linkage disequilibrium to the real causal variant in the mtDNA genome. Third, several variants together located on the same haplotype background predispose to the disease. The role of the pathogenic variants could be additive or multiplicative, or their pathogenic role could occur in a more complex epistatic fashion (e.g. interaction with nuclear factors or with the environment). In the latter two cases, establishing seeming correlation of some macro-haplogroup or some mutation defining such a macro-haplogroup could be irrelevant without considering all haplogroups that are defined by recurrent instances of the suspect mutation and without narrowing the scope to the most basal haplogroups within the suspect macro-haplogroup. In the context of a case–control association study, any of the scenarios above require proper study designs guaranteeing that the statistical association could be true and not spurious, due to artifacts, such as population stratification. Moreover, knowledge of the mtDNA phylogeny is always mandatory in order to interpret the statistical findings with caution. Unfortunately, this knowledge is still limited in most of the studies [7, 9, 48].

The article of Covarrubias et al. [13] represents a prime example of misapplication of the classical SNP-SNP interaction test to mtDNA disease studies, and as the earlier study of Bai et al. [19] it is based on a misconception about haplogroups and the mtDNA phylogeny. None of these studies showing positive associations monitored the possibility of population stratification in their samples (a typical source of spurious false positive associations in complex disease studies), a fact that could be particularly relevant in admixed populations such as ‘African-Americans’ from U.S.A. Unusual frequencies in controls leading to false positive findings have also been reported in regard to other seeming disease variant in other diseases [49]. Even in ‘non-admixed’ populations one could expect specific local patterns of mtDNA composition, so that a control group should have the same number of representatives per micro-region as the case group. It seems that in reality control groups are chosen as convenience samples without monitoring population stratification or as available literature data representing the ‘general population’. Neither way is optimal, so that signals of association are bound to be spurious.

Moreover, most of these studies appear to be statistically underpowered (Table 1). Underpowered means that any positive finding can be explained by chance. The fact that so many underpowered independent studies have pointed to some evidence of association (conflicting in regard to the nucleotide variant involved) can also be seen as the result of publication bias. Only two of the earlier studies have reported negative findings. The Setiawan et al. [18] study could not replicate the Canter’s findings using ‘African-American’ breast cancer women (by way of analyzing a similar sized cohort) and did not find association in another two cohorts or in their joined cohorts analysis; whereas the Mosquera-Miguel et al. [5] study could not replicate any finding using two independent cohorts of European ancestry. It is however paradoxical that the number of cohorts showing negative findings is higher than the number of cohorts showing positive (conflicting) findings (see Table 1).

## Conclusions

The present survey on breast cancer studies probably represents just the tip of the iceberg in case–control mtDNA studies related to complex and multifactorial diseases. Thus for instance, Fachal et al. [50], analyzed several cohorts of Alzheimer, Parkinson and migraine patients, and coupled with a deep review of the large literature in these neurological disorders, arrived to the conclusion that the positive findings of association are largely inconsistent. They also found that from a total of 35 different association studies reviewed, only 11% used replications cohorts, 14% of them provided estimates on statistical power (and the majority were underpowered, as it is the case in the breast cancer studies reviewed here) or, for example, only 17% of their *P*-values computed on hypothesis testing were adjusted for multiple tests (and this correction was not always applied correctly).

We have shown, by way of example concerning mtDNA studies on breast cancer, that most case–control association mtDNA studies (independent of the impact factor of the journal in which they were published) were undertaken in a very relaxed manner, with questionable scientific standards.
